# ﻿*Lysimachia nanshanensis* (Primulaceae), a new species from southwest Hunan, China

**DOI:** 10.3897/phytokeys.262.153293

**Published:** 2025-09-09

**Authors:** Chi Xiong, Xiao Wang, Xiao-Ying Fu, Kang Luo, Ya Huang, Hong-Jing Zhang, Si-Rong Yi

**Affiliations:** 1 Guangxi Key Laboratory of Plant Conservation and Restoration Ecology in Karst Terrain, Guangxi Institute of Botany, Guangxi Zhuang Autonomous Region and Chinese Academy of Sciences, Guilin, 541006, Guangxi, China Guangxi Institute of Botany, Guangxi Zhuang Autonomous Region and Chinese Academy of Sciences Guilin China; 2 Guizhou Xishui National Nature Reserve Management Bureau, Xishui 564000, Guizhou, China Guizhou Xishui National Nature Reserve Management Bureau Xishui China; 3 College of Life Sciences, Guangxi Normal University, Guilin 541006, Guangxi, China Guangxi Normal University Guilin China; 4 Chongqing Key Laboratory of Development and Utilization of Genuine Medicinal Materials in Three Gorges Reservoir Area, Chongqing Three Gorges Medical College, Wanzhou, 404120, Chongqing, China Chongqing Three Gorges Medical College Chongqing China

**Keywords:** Flora of Hunan, morphology, Nanshan National Park, taxonomy

## Abstract

*Lysimachia nanshanensis*, a new species of Lysimachia ser. Paridiformes discovered in Nanshan National Park of southwest Hunan, China, is described and illustrated. Morphologically, this new species most closely resembles *L. sciadantha* in having whorled leaves and terminal umbels but differs from *L. sciadantha* by having terminal umbels without a peduncle, subpapery leaves with obvious black glandular punctation and striation, longer pedicels, and filaments that are nearly free. Results of molecular phylogenetic analysis, based on nuclear ribosomal internal transcribed spacer (nrITS) and two chloroplast DNA fragments (*matK* and *rbcL*) of 34 *Lysimachia* species, showed that *L. nanshanensis* is closely related to *L. sciadantha*. According to the IUCN Red List Categories and Criteria, the new species is assigned to the Vulnerable (VU D1) category.

## ﻿Introduction

*Lysimachia* L. (Linnaeus 1753: 146) constitutes the second largest genus within the family Primulaceae, encompassing approximately 200 species predominantly found in temperate and subtropical regions of the Northern Hemisphere, as well as in certain tropical mountainous areas (Chen and Hu 1979; Hu and Kelso 1996; Marr and Bohm 1997; Hao et al. 2004; Julius et al. 2016). It has been suggested that the genus may have originated in southwestern China (Chen and Hu 1979), where about 160 species have been documented, with several new species identified in recent years (e.g., Mou et al. 2020; Yi 2020; Ju et al. 2021; Yan et al. 2022, 2023; Zhang et al. 2024).

During a botanical survey conducted in Nanshan National Park (Pilot), Hunan, China, we encountered an unidentified species of *Lysimachia* characterized by whorled leaves and terminal umbels that place it within Lysimachia ser. Paridiformes. A thorough examination of herbarium specimens, together with an extensive review of existing literature on the genus (Wu 1965; Chen and Hu 1979; Chen et al. 1989; Hu and Kelso 1996; Shao et al. 2006; Yan and Hao 2012, 2024; Liu et al. 2014; Zhou et al. 2015; Wang et al. 2018; Huang et al. 2020; Liu et al. 2020; Mou et al. 2020; Yi 2020; Ju et al. 2021; Yan et al. 2022, 2023; Zhang et al. 2024), confirmed that this specimen represents a new species of *Lysimachia*. In addition, we investigated the phylogenetic position of the new taxon, which is formally described and illustrated herein.

## ﻿Materials and methods

### ﻿Morphological analyses

Specimens of the newly identified species were collected during a field expedition to Hunan Nanshan National Park in 2019. Morphological descriptions and measurements were obtained from both living specimens and dried herbarium samples housed at CGMC and KUN. Herbarium materials of relevant *Lysimachia* species were examined at CDBI, GZAC, HGAS, KUN, and PE. Furthermore, additional related *Lysimachia* species were analyzed using online images sourced from the Chinese Virtual Herbarium (https://www.cvh.ac.cn/), the Plant Photo Bank of China (https://ppbc.iplant.cn/), the Global Biodiversity Information Facility (https://www.gbif.org/), and JSTOR Global Plants (http://plants.jstor.org/).

### ﻿Genomic DNA extraction and sequencing

Total genomic DNA was extracted from silica-dried leaves using a modified CTAB method (Chen et al. 2014). The DNA samples were sent to Majorbio (http://www.majorbio.com/) for library construction and next-generation sequencing. A paired-end library with an insert size of 350 bp was constructed, and sequencing was performed using the Illumina HiSeq 4000 platform. Approximately 1 Gb of raw reads was generated and subsequently filtered using the FASTX-Toolkit to remove adapter and low-quality reads (http://hannonlab.cshl.edu/fastx_toolkit/download.html). Clean reads were assembled using GetOrganelle v.1.7.7.0 (Jin et al. 2020) to recover ribosomal genome sequences. Complete chloroplast genome and ribosomal genome data were also assembled using GetOrganelle v.1.7.7.0 (Jin et al. 2020). ITS regions were extracted using ITSx v.1.1.3 (Bengtsson-Palme et al. 2013).

### ﻿Phylogenetic analyses

To determine the phylogenetic placement of this species, we extracted three DNA regions (ITS, *matK*, and *rbcL*) from assembled rDNA and complete plastid genome sequences of the new species. Accessions of the new species (GenBank Acc. ITS: PX070370; *matK*: PX115313; *rbcL*: PX115314) and *Lysimachia sciadantha* (GenBank Acc. ITS: PX111650, – ; *mat*K: PX115315, PX115317; *rbcL*: PX115316, PX115318) were sequenced for this study. Additional sequences from *Lysimachia* and related taxa were downloaded from GenBank based on previous studies (Zhang et al. 2012; Zhang et al. 2024) (see Table 1 for details). The final dataset included 73 accessions representing 35 taxa, with 35 taxa belonging to *Lysimachia* as the ingroup, and *Ardisia verbascifolia* Mez selected as the outgroup.

**Table 1.** Voucher information for phylogenetic analyses and GenBank accession numbers.

Taxon	Voucher	Locality	ITS	matK	rbcL
*Lysimachia alfredi*	Hao394	Lianping, Guangdong, China	JN638405	JF954430	JF942344
	Y2009279	Ruyuan, Guangdong, China	JN638406	JF954429	JF942343
*Lysimachia candida*	Y2010016	Tongbai, Henan, China	JF976884	JF954431	JF942345
	Ge2010001	Yangchun, Guangdong, China	JF976885	JF954432	JF942346
*Lysimachia chapaensis*	GBOWS878	Hekou, Yunnan, China	JF976888	JF954435	JF942349
	GBOWS704	Maguan, Yunan, China	JF976889	JF954436	JF942350
*Lysimachia chekiangensis*	Y2009263-2	Longquan, Zhejiang, China	JF976890	JF954437	JF942351
	Y2009263-1	Longquan, Zhejiang, China	JF976891	JF954438	JF942352
*Lysimachia christinae*	Y2009272	Jiangle, Fujian, China	JF976893	JF954440	JF942354
	Y2009235	Shucheng, Anhui, China	JF976895	JF954442	JF942356
	Y2009209	Jiujiang, Jiangxi, China	JF976896	JF954443	JF942357
*Lysimachia clethroides*	Hao955	Wuxi, Chongqing, China	JF976897	JF954445	JF942359
	Y2009248	Lin’an, Zhejiang, China	JF976898	JF954446	JF942360
	Y2009157	Tongbai, Henan, China	JF976899	JF954448	JF942362
*Lysimachia congestiflora*	GBOWS262	Malipo, Yunnan, China	JF976902	JF954451	JF942365
	Y2009266	Longquan, Zhejiang, China	JF976903	JF954452	JF942366
	Y2009196	Xinjian, Jiangxi, China	JF976904	JF954453	JF942367
*Lysimachia crispidens*	Y2010029	Xinhua, Hubei, China	JF976905	JF954454	JF942368
	Hao212	Yichang, Hubei, China	JF976906	JF954455	JF942369
*Lysimachia danxiashanensis*	DNPC-3711	Danxiashan, Guangdong, China	OR665389	—	PP025352
	DNPC-3711	Danxiashan, Guangdong, China	OR665390	—	PP025354
*Lysimachia decurrens*	Ye et al. 3980	Lianshan, Guangdong, China	JF976907	JF954456	JF942370
	GBOWS1234	Hekou, Yunnan, China	JF976908	JF954457	JF942371
Lysimachia deltoidei var. cinerascens	GLM081121	Zhongdian, Yunnan, China	JF976909	JF954458	JF942372
	Hao731	Yongsheng, Yunnan, China	JF976910	JF954459	JF942373
	Hao & Yan1033	Dali, Yunnan, China	JF976911	JF954460	JF942374
*Lysimachia dextrorsiflora*	Y2009265-2	Longquan, Zhejiang, China	JF976912	JF954461	JF942375
	Y2009265-1	Longquan, Zhejiang, China	JF976913	JF954462	JF942376
*Lysimachia erosipetala*	Y2010037-2	Emeishan, Sichuan, China	JF976914	JF954463	JF942377
	Y2010037-1	Emeishan, Sichuan, China	JF976915	JF954464	JF942378
Lysimachia fistulosa var. wulingensis	Ning20101	Jinggangshan, Jiangxi, China	JF976916	JF954466	JF942380
	Ye et al. 3561	Lianshan, Guangdong, China	JF976917	JF954467	JF942381
*Lysimachia fordiana*	Y2009285	Ruyuan, Guangdong, China	JF976919	JF954469	JF942383
	Ye et al. 3940	Lianshan, Guangdong, China	JF976920	JF954470	JF942384
*Lysimachia grammica*	Hao209	Wuhan, Hubei, China	AF547691	JF954478	JF942392
*Lysimachia hemsleyana*	Y2010008	Tongbai, Henan, China	JF976928	JF954480	JF942394
	Y2009245	Lin’an, Zhejiang, China	JF976929	JF954481	JF942395
	Guo20001	Ningguo, Anhui, China	JF976932	JF954484	JF942398
*Lysimachia hemsleyi*	Hao730	Yongsheng, Yunnan, China	JF976934	JF954487	JF942401
	Hao713	Huili, Sichuan, China	JF976935	JF954488	JF942402
*Lysimachia heterogenea*	Y2010009	Tongbai, Henan, China	JF976938	JF954491	JF942405
	Y2009199	Jiujiang, Jiangxi, China	JF976939	JF954493	JF942407
*Lysimachia klattiana*	Y2010014-2	Tongbai, Henan, China	JF976946	JF954500	JF942414
*Lysimachia kwangtungensis*	DNPC-3743	Danxiashan, Guangdong, China	OR941025	—	PP025355
	Y2010014-1	Tongbai, Henan, China	JF976947	—	JF942415
*Lysimachia lobelioides*	Y2010001	Jingping, Yunan, China	JF976950	JF954504	JF942418
	Hao303	Menglian, Yunnan, China	JF976951	JF954505	JF942419
*Lysimachia longipes*	Guo XH 200012	Shitai, Anhui, China	JF976952	JF954506	JF942420
	Y2009255-2	Kaihua, Zhejiang, China	JF976953	JF954507	JF942421
	Y2009255-1	Kaihua, Zhejiang, China	JF976954	JF954508	JF942422
*Lysimachia melampyroides*	Deng YF 15945	Xinning, Hunan, China	JF976955	JF954509	JF942423
	Lichanghan8174	Shangzhi, Hunan, China	JF976956	JF954510	JF942424
*Lysimachia nanshanensis*	XC25023	Chengbu, Hunan, China	PX070370	PX115313	PX115314
*Lysimachia omeiensis*	Hao224	Emeishan, Sichuan, China	JF976957	JF954511	JF942425
	Y2010033	Emeishan, Sichuan, China	JF976958	JF954512	JF942426
*Lysimachia paridiformis*	GXJ042	China	JF976959	JF954513	JF942427
Lysimachia paridiformis var. stenophylla	Deng YF 15921	China	MG877820	MG950486	MG950592
	Y2010044	Emeishan, Sichuan, China	JF976962	JF954516	JF942430
*Lysimachia patungensis*	Ye et al. 3851	Lianshan, Guangdong, China	JF976964	JF954518	JF942432
	Y2009280	Ruyuan, Guangdong, China	JF976965	JF954519	JF942433
	Y2009258	Kaihua, Zhejiang, China	JF976966	JF954520	JF942434
	Y2009187	Jinggangshan, Jiangxi, China	JF976967	JF954521	JF942435
*Lysimachia pentapetala*	Y2010013-1	Tongbai, Henan, China	JN638407	JF954523	JF942437
*Lysimachia phyllocephala*	GLM07662	Yanjin, Yunnan, China	JF976933	JF954485	JF942399
	Y2010048	Nanchuan, Chongqing, China	JF976968	JF954524	JF942438
	Y2010030	Emeishan, Sichuan, China	JF976969	JF954525	JF942439
*Lysimachia pseudohenryi*	Guo XH 20007	China	MG877828	—	MG950600
*Lysimachia rubiginosa*	Hao419	Dujiangyan, Sichuan, China	JF976972	JF954528	JF942442
	Y2010036	Emeishan, Sichuan, China	JF976973	JF954529	JF942443
	Hao704	Hongya, Sichuan, China	JF976974	JF954530	JF942444
*Lysimachia sciadantha*	XC25025	Jiangjin, Chongqing, China	—	PX115315	PX115316
	WX2025011	Xishui, Guizhou, China	PX111650	PX115317	PX115318
*Ardisia verbascifolia*	GBOWS1216	Hekou, Yunnan, China	JN638408	JN638409	JN638410

All sequences were aligned using MAFFT, and aligned regions were concatenated using PhyloSuite v.1.2.3 (Katoh and Standley 2013; Zhang et al. 2020). The best substitution models—SYM+G4 for ITS and GTR+F+I+G4 for *matK* and *rbcL*—were selected using ModelFinder (Kalyaanamoorthy et al. 2017), with the corrected Akaike Information Criterion (AICc). Bayesian inference (BI) analysis was performed in MrBayes within PhyloSuite v.1.2.3 (Ronquist et al. 2012; Zhang et al. 2020). The Markov chains were run for 1,000,000 generations, with sampling every 1,000 generations and discarding the first 25% as burn-in. Maximum likelihood (ML) analysis was conducted using IQ-TREE with 1,000 bootstrap replicates in PhyloSuite v.1.2.3 (Guindon et al. 2010; Minh et al. 2013; Nguyen et al. 2015; Zhang et al. 2020).

## ﻿Results

**Figure 1.**
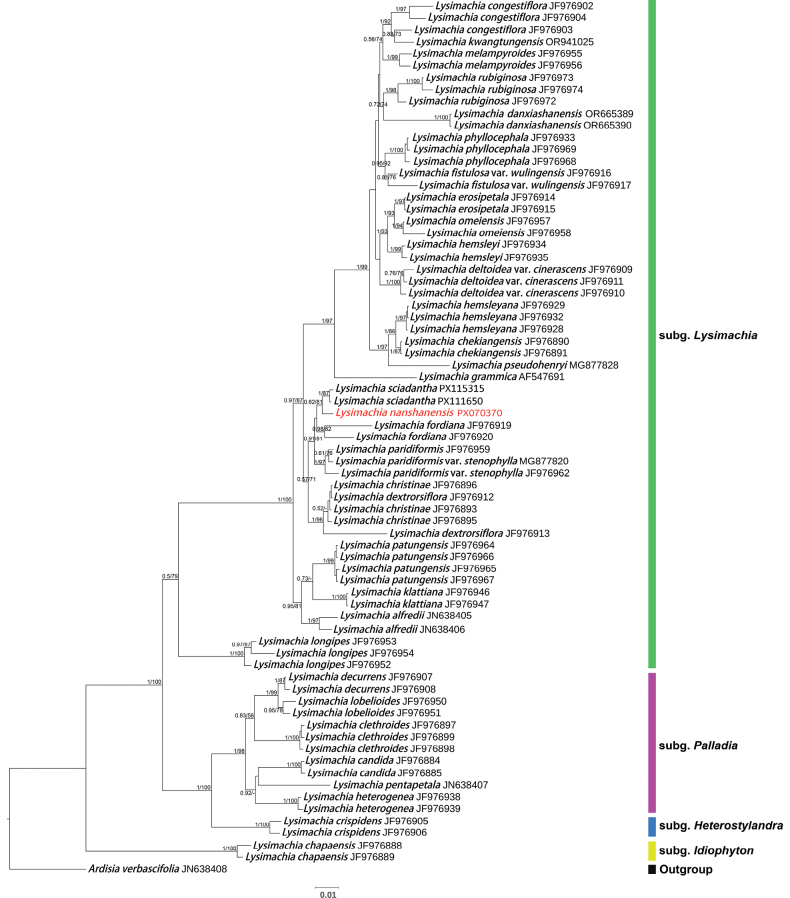
Bayesian tree from analysis of combined nrITS and two-plastid (*mat*K and *rbc*L) marker data of 34 species of *Lysimachia*. The posterior probabilities (PP) of BI and bootstrap values (BS) of ML are listed at each node. The new species is highlighted in red.

The aligned ITS, *matK*, and *rbcL* datasets were 655 bp, 711 bp, and 615 bp, respectively, yielding a concatenated alignment of 1,981 bp. The resulting phylogenetic tree (Fig. 1) was largely congruent with previous studies (Zhang et al. 2012; Zhang et al. 2024). The new species and *L. sciadantha* C. Y. Wu formed a monophyletic pair (PP/BS = 0.82/81) sister to *L. fordiana* Oliv., *L. paridiformis* Franch., and L. paridiformis var. stenophylla Franch. (PP/BS = 0.91/81) (Fig. 1).

## ﻿Taxonomic treatment

### ﻿ Lysimachia nanshanensis

Taxon classificationPlantaeEricalesPrimulaceae

C.Xiong & S.R.Yi sp. nov.

8BA0674D-5124-5823-BB85-A8D971729B2A

urn:lsid:ipni.org:names:77368925-1

Figs 2, 3

#### Diagnosis.

*Lysimachia nanshanensis* belongs to ser. Paridiformes F.H.Chen & C.M.Hu (Chen and Hu 1979: 36) and is similar to *L. sciadantha* C.Y. Wu (Wu 1965: 53) by sharing some morphological characters, such as the middle and lower leaves bract-like and opposite, upper leaves clustering at the stem top, flowers densely on umbel. But differs from *L. sciadantha* in leaves 4–6 (vs. 4) in a whorl, subpapery (vs. stiffly papery), elliptic, ovate to obovate (vs. elliptic), apex obtuse to mucronate (vs. acute to short acuminate), umbels terminal without peduncle (vs. peduncle 1.8–6 cm long), pedicel 3–6 cm (vs. 1.2–2.5) long, filaments almost free (vs. base tube 3–3.5 mm).

#### Type.

China • Hunan: Chengbu, Nanshan National Park (南山国家公园), Yu’nüxi (玉女溪), 20 April 2019, 26°11'05"N, 110°11'38"E, elev. 750 m, on the rock wall of the valley, *Si-Rong Yi YSR8168* (holotype, KUN!; isotypes, KUN!, CGMC!).

#### Description.

Perennial herbs. Rhizome short, with many roots up to 15 cm long. Stems erect, often 3–4-noded, up to 30 cm long, pale pink when young. Leaves opposite, lower and middle ones bract-like; upper ones 4–6 in a terminal whorl, subpapery, with obviously black glandular punctate and striate, elliptic, ovate to obovate, 4.5–11 × 2.0–4.5 cm, petiole 4–8 mm long and pale purple; leaf blades apex obtuse to mucronate, base cuneate and descending into petiole, margin entire to shallowly undulate; blades adaxially pale green, densely light purple short glandular stripes, abaxially pale greenish; veins sunken adaxially, protuberant abaxially, lateral veins 4–6 pairs. Cauline bracts ovate-lanceolate, 5–11 × 2.5–4 mm. Umbels terminal, many flowers; pedicel slender, 3–6 cm long, pale green, sparsely coated, peduncle absenced. Calyx 5-lobed, pale purple brownish to green purple; lobes lanceolate, entire, glabrous, 6.5–7.5 mm long, 1.5–1.8 mm wide in middle, apex acuminate, abaxially sparsely light purple short glandular stripes. Corolla broad campaniform to trumpet-shaped, yellow; tube 0.3–0.5 mm, lobes 9–13 × 3.5–4.5 mm, apex undulated to 2–3-lobed shallowly. Stamens 5, subequal, yellow, 5–6 mm long, filaments almost free, often curved, slightly farinose; anthers elliptic, dorsifixed, 1.2–1.5 mm long, 0.5–0.6 mm wide, opening by lateral slits. Ovary globose, glabrous,1.2–1.5 mm in diam., style 3.6–4.5 mm, erect. Capsule globose, 1.6–2 mm in diam., style persistent.

**Figure 2.**
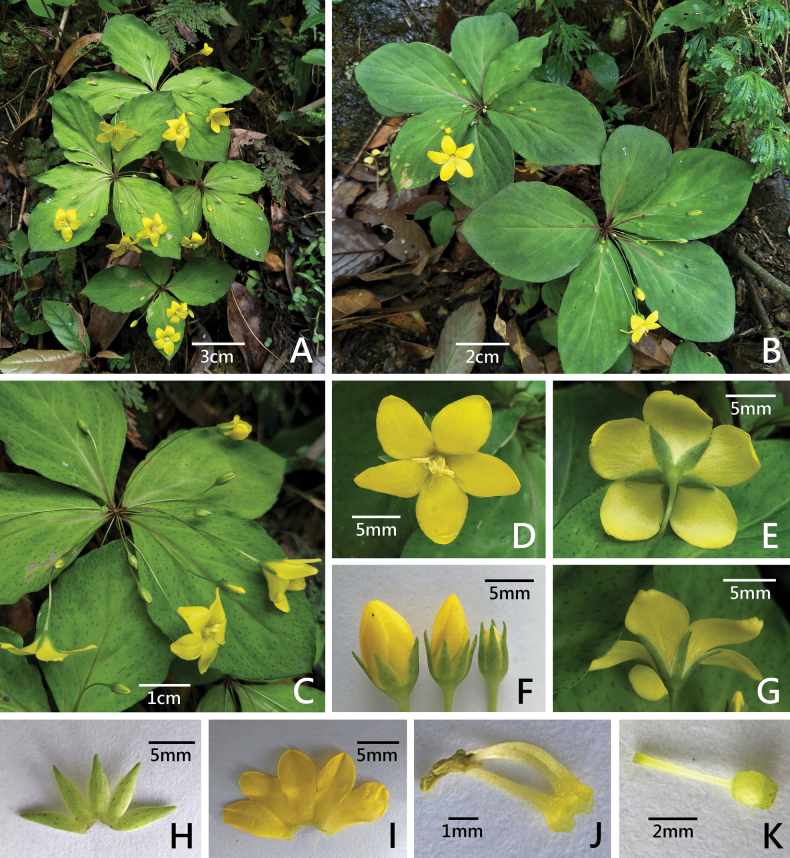
*Lysimachia nanshanensis* C.Xiong & S.R.Yi. A, B. Habit; C. Terminal umbels; D. Flower (front view); E. Flower (back view); F. Flower buds; G. Flower (side view); H. Abaxial of calyx; I. Adaxial side of corolla; J. Stamens; K. Pistil.

#### Phenology.

Flowering from April to May and fruiting from July to August.

#### Etymology.

The specific epithet ‘*nanshanensis*’ refers to the type locality where the new species was found, Nanshan National Park, Southwest Hunan. The Chinese name is “南山落地梅 (nán shān luò dì méi)”.

**Figure 3.**
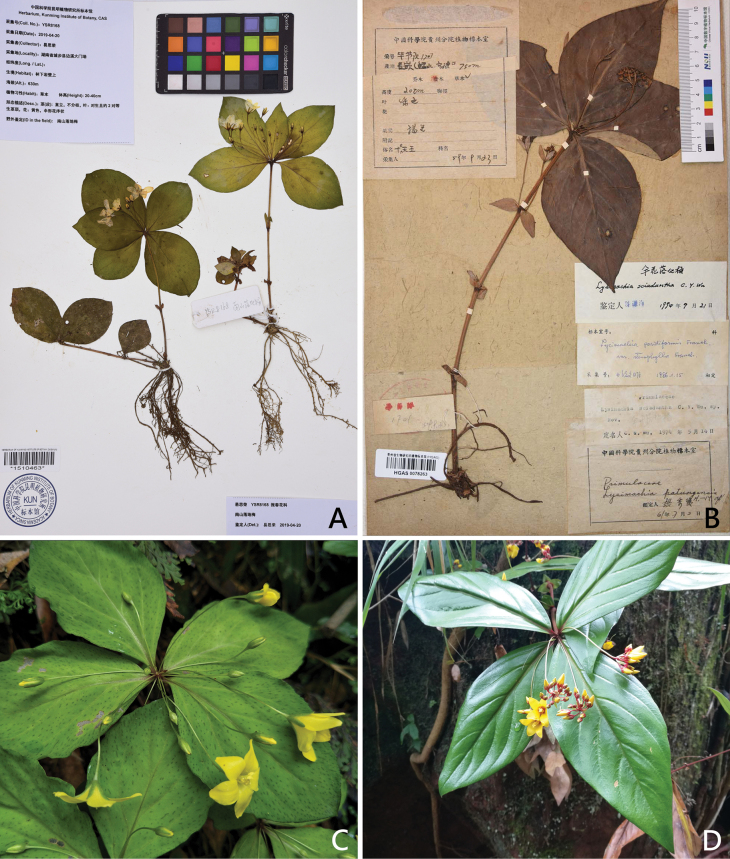
*Lysimachia nanshanensis* and *L. sciadantha*. A. Holotype of *L. nanshanensis* (KUN1510463); B. Isotype of *L. sciadantha* (HGAS0078263); C. Habit of *L. nanshanensis*; D. Habit of *L. sciadantha* (D photographed by Hua-An Zhang).

#### Distribution and habitat.

**Figure 4.**
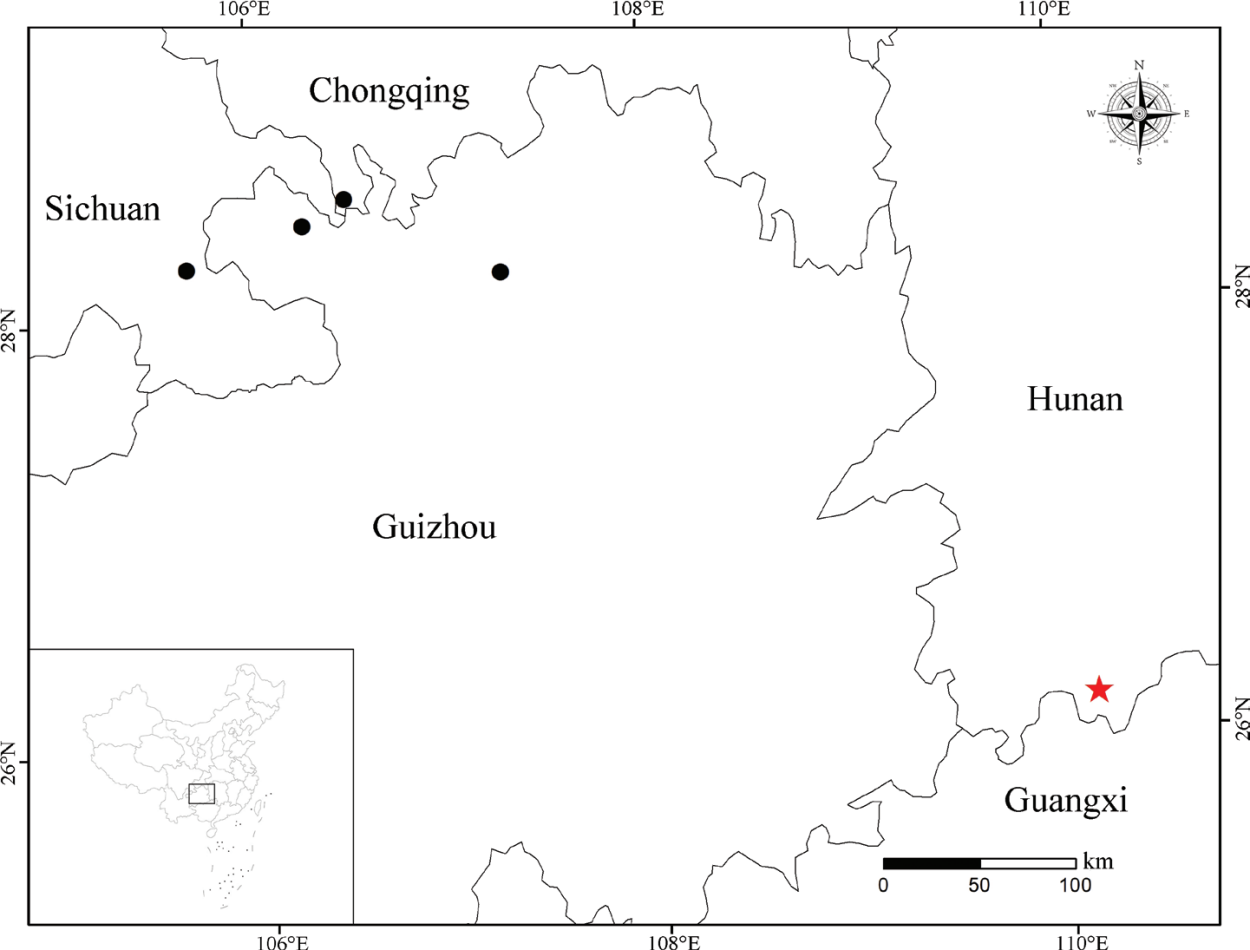
Distribution of *Lysimachia nanshanensis* (red star) and *L. sciadantha* (black circle).

*Lysimachia nanshanensis* C.Xiong & S.R.Yi was found only in Yu’nüxi and Shilipingtan, Nanshan National Park, Chengbu County, Hunan, China (Fig. 4). It grows on rocky cliffs within the valley at elevations of 750–1200 m.

#### Additional specimens examined.

*Lysimachia sciadantha*: China • Guizhou Province, Xishui, Changqian’gou, *Bijie expedition 1701* (HGAS0078263, GZAC0018115), Suiyang, Kuankuoshui, *Yi Li 9306318* (ZY0030002); • Chongqing Municipality, Jiangjin, Simian Mountain, *Z.Y. Liu 183194* (PE01861962); • Sichuan Province, Xuyong, *X.F. Gao et al. HGX10826* (CDBI0234655), *X.F. Gao et al. HGX11693B* (CDBI0226619).

#### Conservation assessment.

*Lysimachia nanshanensis* is currently known from the type locality, consisting of approximately three populations with 800 mature individuals. The estimated extent of occurrence (EOO) and area of occupancy (AOO) of the new species are approximately 30,000 m^2^ and 1200 m^2^, respectively. This species grows on rocky cliffs in the valley, an area largely undisturbed by human activity. Furthermore, its distribution falls within Nanshan National Park, affording it a degree of natural protection. Based on the IUCN Red List Categories and Criteria (IUCN 2025), the species is temporarily assessed as Vulnerable (VU D1).

## ﻿Discussion

Lysimachia ser. Paridiformes comprises two species and one variety: *Lysimachia sciadantha*, *L. paridiformis* Franch., and L. paridiformis var. stenophylla Franch. (Franchet 1884: 443). These taxa share several morphological traits, including four or more whorled leaves at the stem apex, the degeneration of lower leaves into scale-like structures, a sparse distribution of black glandular dots on the plant body, the formation of umbel-like flower clusters at the stem terminus, and the fusion of the lower portions of the filaments into a tubular structure. *L. nanshanensis* exhibits a close relationship with *L. sciadantha*, primarily due to the elongated nature of its pedicels or peduncles (Fig. 3). Additionally, *L. nanshanensis* resembles *L. paridiformis* and L. paridiformis var. stenophylla; however, the latter two are characterized by much shorter pedicels, facilitating their differentiation. A detailed comparison of the related species is provided in Table 2.

**Table 2.** Morphological comparisons of *Lysimachia nanshanensis*, *L. sciadantha*, *L. paridiformis* and L. paridiformis var. stenophylla.

Characters	L. nanshanensis	L. sciadantha	L. paridiformis	L. paridiformis var. stenophylla
height	up to 30 cm	30–40 cm	10–45 cm	up to 45 cm
leaves	4–6 in a whorl	4 in a whorl	4–6 in a whorl	6–18 in a whorl
texture of dried leaves	subpapery	stiffly papery	papery to stiffly papery	papery to stiffly papery
leaf blades	elliptic, ovate to obovate, 4.5–11 × 2.0–4.5 cm, base cuneate to round, apex obtuse to mucronate	elliptic, 10–14 × 6–7.5 cm, base broadly cuneate, apex acute to short acuminate	broadly obovate to elliptic, 5–17 × 3–10 cm, base cuneate, apex short acuminate	narrowly elliptic to lanceolate, 4–16 × 1.2–5 cm, base cuneate, apex acuminate
glands on leaf surface	with obviously black glandular punctate and striate	with slightly raised scattered transparent glandular stripes	with or without black glandular stripes	usually with black glandular striate
peduncle	absenced	1.8–6 cm	absenced	absenced
pedicel	3–6 cm	1.2–2.5 cm	0.5–1.5 cm	up to 3 cm
filaments	almost free	base tube 3–3.5 mm high	base tube ca. 2 mm high	base tube 2–3 mm high

According to the “Flora of China” (Chen et al. 1989; Hu and Kelso 1996), *L. fordiana* was originally classified within ser. Fordianae Chen & C. M. Hu. However, molecular phylogenetic studies demonstrate that this species forms a well-supported clade with members of ser. Paridiformes. Morphologically, *L. fordiana* shares several characteristics with *L. paridiformis*, particularly in having densely arranged upper leaf pairs that appear verticillate and in bearing terminal, abbreviated, nearly capitate racemes. The most conspicuous diagnostic feature distinguishing *L. fordiana* from *L. paridiformis* is its significantly larger and thicker leaves.

Regarding their geographical distribution, *L. paridiformis* and L. paridiformis var. stenophylla are widely distributed across several regions, mainly in Guizhou, Chongqing, Sichuan, Hunan, Hubei, Guangxi, Guangdong, and Yunnan in China (Chen et al. 1989; Hu and Kelso 1996). *L. fordiana* is primarily found in lower-latitude regions of China, including Yunnan, Guangdong, and Guangxi (Chen et al. 1989; Hu and Kelso 1996). In contrast, *L. sciadantha* occurs in a limited number of counties at the confluence of Guizhou, Chongqing, and Sichuan provinces. Currently, *L. nanshanensis* is known only from Chengbu County in southeastern Hunan (Fig. 4).

## Supplementary Material

XML Treatment for Lysimachia nanshanensis

## ﻿Additional information

### Use of AI

No use of AI was reported.

### Author ORCIDs

Chi Xiong https://orcid.org/0000-0002-9082-2539

Xiao Wang https://orcid.org/0009-0006-1253-4095

Xiao-Ying Fu https://orcid.org/0009-0009-1090-877X

Kang Luo https://orcid.org/0009-0006-5402-5141

Ya Huang https://orcid.org/0000-0003-3939-4046

Hong-Jing Zhang https://orcid.org/0000-0003-0025-9269

Si-Rong Yi https://orcid.org/0000-0002-3883-6175

### Data availability

All of the data that support the findings of this study are available in the main text.
